# Clinical and genetic spectrum of cytochrome P450 oxidoreductase deficiency in Chinese patients

**DOI:** 10.1530/EC-26-0247

**Published:** 2026-09-08

**Authors:** TingTing Wang, Chong Li, Chao Han, YanYan Zhao, GuangZhao Qi, LiLi Zheng

**Affiliations:** ^1^ Department of Endocrinology and Metabolism, The First Affiliated Hospital of Zhengzhou University, Zhengzhou, Henan, China; ^2^ Department of General Medicine, The First Affiliated Hospital of Zhengzhou University, Zhengzhou, Henan, China; ^3^ Department of Pharmacy, The First Affiliated Hospital of Zhengzhou University, Zhengzhou, Henan, China

**Keywords:** cytochrome P450 oxidoreductase, PORD, WBBC, gnomAD, genetic prevalence

## Abstract

**Background:**

Cytochrome P450 oxidoreductase deficiency (PORD) is a rare autosomal recessive disorder with heterogeneous endocrine, reproductive, DSD-related, and skeletal manifestations. Population-specific data in Chinese patients remain limited.

**Methods:**

We described two Chinese probands with genetically confirmed PORD and systematically reviewed published Chinese cases. Clinical features, POR genotypes, skeletal manifestations, adrenal function, DSD phenotypes, and reproductive features were extracted. Carrier frequency and modeled genetic prevalence were estimated using gnomAD and WBBC, with variants stratified by evidence of pathogenicity.

**Results:**

The pooled cohort included 40 unique Chinese patients, comprising two newly reported probands and 38 literature-derived cases. One proband carried p.R457H and a novel frameshift variant, p.N178Efs*28; the second carried p.R457H and p.Y607C. The recurrent p.R457H variant accounted for 40 of 80 alleles in the Chinese cohort. Using the expanded exploratory variant set, the modeled genetic prevalence was 2.4 per 1,000,000 individuals in WBBC Chinese and 3.3 per 1,000,000 individuals in gnomAD East Asians.

**Conclusion:**

Chinese patients with PORD show broad clinical heterogeneity, and p.R457H is a recurrent East Asian-enriched POR variant. The novel p.N178Efs*28 variant expands the POR mutational spectrum. Population-database estimates should be interpreted as modeled genetic prevalence rather than observed clinical incidence, especially when predicted deleterious variants are included.

## Background

Cytochrome P450 oxidoreductase (POR) deficiency (PORD) (OMIM: 613571 and 2015750) is a rare autosomal recessive disorder caused by biallelic pathogenic variants in POR. PORD is considered a rare form of congenital adrenal hyperplasia (CAH). However, unlike classic 21-hydroxylase deficiency, it may present with a complex combination of impaired steroidogenesis, disorders/differences of sex development (DSD), skeletal malformations, menstrual disturbance, infertility, and variable adrenal dysfunction (1). POR transfers electrons from reduced nicotinamide adenine dinucleotide phosphate (NADPH) to multiple microsomal cytochrome P450 enzymes, including CYP17A1, CYP19A1, and CYP21A2, which are involved in steroidogenesis; CYP51A1, which is involved in cholesterol synthesis; CYP26 enzymes, which regulate retinoic acid metabolism; and CYP3A4, which contributes to drug metabolism. This broad role of POR explains why PORD can affect endocrine, reproductive, skeletal, developmental, and pharmacological phenotypes. Homozygous null mutations are lethal in knockout mouse models (2, 3). In humans, disease severity is thought to depend on the residual activity of the affected POR variants and the extent to which individual P450-dependent pathways are impaired (4).

Since PORD was first described as a distinct clinical entity in 2004, more than 100 patients have been reported worldwide, although the true number is likely underestimated because mild or atypical cases may remain undiagnosed (5). The PORD phenotype is heterogeneous and may include glucocorticoid deficiency, abnormal steroid precursor accumulation, maternal virilization during pregnancy, DSD, and skeletal defects involving the cranium, face, hands and feet, large joints, long bones, spine, or thorax. Severe cases overlap with Antley–Bixler syndrome, whereas milder or non-classic cases may present later with menstrual irregularity, ovarian cysts, infertility, or biochemical abnormalities without obvious skeletal malformation. Because clinical presentation is variable and not always pathognomonic, PORD may be missed or misdiagnosed as other forms of CAH, polycystic ovary syndrome, isolated reproductive dysfunction, or skeletal dysplasia. Diagnosis is usually confirmed by molecular genetic testing, supported by steroid profiling and assessment of adrenal reserve where clinically indicated. Treatment is individualized and may include glucocorticoid replacement or suppression therapy, stress-dose education in patients with impaired adrenal reserve, management of DSD or skeletal complications, and reproductive counseling.

The clinical prevalence of PORD remains uncertain. Traditionally, prevalence estimates for rare diseases have relied on clinically diagnosed cases; however, this approach is limited for PORD because of its rarity, phenotypic heterogeneity, variable age at diagnosis, and possible ascertainment bias toward severe pediatric or skeletal presentations (6). Large-scale population genomic databases now provide an opportunity to estimate carrier frequency and modeled genetic prevalence for rare autosomal recessive disorders (7, 8). In this approach, allele frequencies of disease-associated variants are aggregated and used to estimate expected disease prevalence under Hardy–Weinberg equilibrium assumptions (9). This method was demonstrated to have a strong correlation between the estimation and the respective clinically observed disease frequencies in rare diseases with a known prevalence (10). However, such estimates depend strongly on variant classification. In particular, rare missense variants predicted to be damaging by *in silico* tools should not be considered equivalent to ACMG/AMP-classified pathogenic or likely pathogenic variants. Therefore, population-based estimates should distinguish known pathogenic variants from predicted deleterious variants and should be interpreted as modeled genetic prevalence rather than observed clinical incidence.

The Genome Aggregation Database (gnomAD) provides population-scale allele frequency data from unrelated individuals ascertained largely without severe pediatric disease and includes multiple ancestry groups, including African/African American, Ashkenazi Jewish, East Asian, Finnish, Latino/Admixed American, non-Finnish European, South Asian, and other/unspecified populations (7). The Westlake BioBank for Chinese (WBBC) pilot project includes 4,535 whole-genome sequencing samples and 5,841 high-density genotyping samples from Chinese individuals, providing a population-specific resource for assessing rare variants in China (8). Because gnomAD and WBBC differ in sample composition, sequencing strategy, coverage, and ancestry structure, comparisons between these databases require cautious interpretation. In this study, we report two Chinese probands with genetically confirmed PORD, including one patient carrying a novel POR frameshift variant, c.531dup:p.N178Efs*28. We also performed a systematic review of published Chinese PORD cases and analyzed the clinical and genetic characteristics of 40 unique Chinese patients, comprising 38 literature-derived cases and two newly reported cases. Finally, we estimated POR carrier frequency and modeled genetic prevalence using gnomAD and WBBC, with attention to the distinction between established pathogenic/likely pathogenic variants and predicted deleterious variants.

## Methods

### Clinical examination and case reporting

The probands with genetically confirmed PORD were recruited from the First Affiliated Hospital of Zhengzhou University. The study was conducted in accordance with the Declaration of Helsinki (2013 revision) and was approved by the Ethics Committee of the First Affiliated Hospital of Zhengzhou University. Written informed consent was obtained from the patient or the patient’s guardian for the collection of clinical data and for the publication of potentially identifiable clinical photographs, radiographs, and accompanying clinical information.

The two newly reported cases were described in accordance with the CARE case-reporting principles, including patient information, clinical findings, diagnostic assessment, genetic testing, treatment, follow-up, and outcome. Clinical examination included assessment of skeletal abnormalities, sex development, growth pattern, pubertal development, menstrual history, reproductive history, and adrenal function. Physical examination, skeletal survey, and X-ray radiography were performed for skeletal assessment. For assessment of sex development, external genital findings were recorded where available, including clitoral size, labial fusion, urogenital openings, and history of genital surgery. Internal genital structures and adrenal morphology were assessed by ultrasonography and/or enhanced computed tomography.

Height and weight were evaluated according to the longitudinal Chinese growth standards. Serum hormone concentrations and corresponding reference ranges were collected from laboratory records, including progesterone, pregnenolone, 17-hydroxyprogesterone, corticosterone, deoxycorticosterone, dehydroepiandrosterone or dehydroepiandrosterone sulfate, cortisol, aldosterone, androstenedione, testosterone, estradiol, luteinizing hormone, follicle-stimulating hormone, adrenocorticotropic hormone, renin, serum sodium, serum potassium, and blood pressure, where available. ACTH stimulation test results were recorded when the test was performed. If ACTH stimulation testing was not performed before glucocorticoid treatment, this was explicitly recorded and taken into account when interpreting adrenal reserve.

### Whole-genome sequencing and Sanger validation

Peripheral blood samples were collected from the proband and her parents. Genomic DNA was extracted from peripheral blood leukocytes and used for library preparation with the Illumina TruSeq DNA Sample Preparation Kit according to the manufacturer’s instructions. The libraries were sequenced on the Illumina HiSeq 2500 platform. Reads that met quality-control requirements were aligned to the human reference genome GRCh37/hg19 from the UCSC Genome Browser. Candidate variants in POR were identified from the sequencing data and interpreted in relation to the clinical phenotype and inheritance pattern.

The identified POR variants in the proband were validated by Sanger sequencing. Parental Sanger sequencing was performed to determine whether the variants were inherited in trans. Variant nomenclature followed Human Genome Variation Society recommendations. The novel frameshift variant c.531dup:p.N178Efs*28 was assessed according to ACMG/AMP variant interpretation criteria, including predicted loss-of-function effect, population frequency, segregation evidence, and phenotype consistency.

### Identification of published Chinese patients with PORD

Published case reports and case series of Chinese patients with PORD were identified by searching CNKI, WANFANG, PubMed, and Web of Science on October 15, 2024. The following search terms were used: ‘P450 oxidoreductase’ AND (‘deficiency’ OR ‘deficient’ OR ‘mutation’) AND (‘Chinese’ OR ‘China’). The literature search, screening, eligibility assessment, and inclusion process were conducted and reported with reference to the PRISMA 2020 principles.

Eligible reports included patients of Chinese ancestry with biallelic POR variants and extractable clinical information. Patients were excluded if they had only one reported POR variant, insufficient clinical information, variants considered inconsistent with monogenic PORD because of high population frequency, or duplicate reporting across publications. Duplicate cases were identified by comparing sex, age at diagnosis, hospital or region, year of publication, clinical phenotype, POR genotype, family history, and other case-specific information across Chinese- and English-language reports (11, 12, 13, 14, 15, 16, 17, 18, 19, 20, 21, 22, 23, 24, 25, 26, 27, 28, 29, 30, 31, 32, 33, 34, 35, 36, 37, 38).

The numbers of records identified, records excluded, full-text articles assessed, patients excluded, and final patients included are summarized in a flow diagram adapted from the PRISMA 2020 framework. The final pooled cohort consisted of two newly reported probands and eligible literature-derived Chinese patients.

### Data extraction from published cases

Clinical and genetic data were manually extracted from the included case reports and case series. Extracted variables included sex, karyotype, age at diagnosis, age group at presentation, maternal virilization during pregnancy, external genital phenotype, internal genital findings, menstrual or reproductive manifestations, adrenal insufficiency, glucocorticoid treatment, skeletal abnormalities, POR genotype, and reported variant interpretation.

For each clinical variable, the denominator was defined as the number of patients with available information. Missing or unreported features were recorded as unavailable rather than absent. Therefore, percentages were calculated using available-case denominators. For example, adrenal insufficiency was calculated as the number of patients with reported adrenal insufficiency divided by the number of patients with available adrenal-function information.

### Skeletal malformation assessment and genotype grouping

The severity of skeletal malformations was assessed using a calculated skeletal malformation score for each patient. The score was adapted from the skeletal malformation scoring approach described by Krone *et al.* and included reported abnormalities of the craniofacial region, hands and feet, large joints, spine, thorax, and long bones. Features that were not mentioned in the original case report were treated as missing rather than normal.

For genotype–phenotype analysis, patients were grouped according to POR genotype as follows: group A, R457H/R457H; group B, R457H/null; group C, R457H/other missense or in-frame variant; and group D, other/null or other/other. Null variants were defined as frameshift, nonsense, canonical splice-site, or other variants predicted to cause major loss of function. Because the skeletal score was derived from retrospective case reports and has not been independently validated in this Chinese cohort, genotype–phenotype comparisons were considered exploratory.

### Population databases

Compressed variant call format files from gnomAD v2.1.1 based on GRCh37/hg19 were obtained from the gnomAD database on March 15, 2023. The dataset included 125,748 exome sequences and 15,708 whole-genome sequences from 141,456 individuals. Population-specific variant allele frequencies were extracted for the following ancestry groups: African/African American, Ashkenazi Jewish, East Asian, Finnish, Latino/Admixed American, non-Finnish European, South Asian, and other/unspecified ancestry.

The Westlake BioBank for Chinese pilot project was also analyzed. WBBC includes 4,535 whole-genome sequencing samples and 5,841 high-density genotyping samples from Chinese individuals. Variant call format files were downloaded from the WBBC website on May 27, 2023. The files contained allele count, allele number, and homozygote information for each variant.

Only high-quality variants were retained for downstream analysis. Variants were included if they were labeled ‘PASS’ and were covered in more than 50% of individuals in the corresponding dataset. Because gnomAD and WBBC differ in sample size, sequencing strategy, variant discovery approach, coverage, and ancestry structure, comparisons between databases were interpreted with caution.

### Variant annotation and classification

All POR variants identified in gnomAD and WBBC were annotated using ClinVar version 20230315 and relevant published literature. Variants annotated as benign or likely benign were excluded from prevalence modeling. Variants annotated as pathogenic or likely pathogenic in ClinVar or classified as pathogenic/likely pathogenic in published reports according to ACMG/AMP criteria were included in the conservative variant set.

To address uncertainty in variant interpretation, variants were stratified into three nested groups for sensitivity analysis:

Set 1, conservative set: ClinVar pathogenic/likely pathogenic variants and published ACMG/AMP pathogenic/likely pathogenic POR variants.

Set 2, moderate set: Set 1 plus rare high-confidence predicted loss-of-function variants, including nonsense, frameshift, start-loss, and canonical splice-site variants predicted to cause major loss of function.

Set 3, expanded exploratory set: Set 2 plus rare missense and in-frame indel variants predicted to be deleterious by multiple computational tools.

Variants with one or more homozygotes in population databases or variant allele frequency greater than 0.005 were excluded from candidate disease-causing variant sets, unless strong published evidence supported pathogenicity. LOFTEE was used to annotate high-confidence predicted loss-of-function variants. Missense variants of uncertain significance were assessed using SIFT (39), PolyPhen-2 (40), CADD (41), REVEL (42), and MetaLR (43). Missense variants were not classified as pathogenic solely on the basis of computational prediction. Instead, they were designated as predicted deleterious variants and included only in the expanded exploratory analysis.

In-frame indel variants were considered candidate deleterious variants only if they were rare, located in functionally relevant POR regions, and affected regions in which pathogenic or likely pathogenic variants had previously been reported. All variant classifications used for prevalence modeling were listed in Supplementary Table S1 (see section on Supplementary materials given at the end of the article), with separate columns indicating ClinVar classification, ACMG/AMP evidence, prediction-tool results, population frequency, homozygote count, and assignment to Set 1, Set 2, or Set 3.

### Carrier frequency and modeled genetic prevalence estimation

Carrier frequency and modeled genetic prevalence were calculated separately for each variant set. Variant allele frequencies of selected POR variants were aggregated within each population. The aggregated candidate disease-allele frequency, denoted as *Q*, was calculated as:

*Q* = 1 − Π*ᵢ*_= 1_^*n*^(1 − VAF*ᵢ*)

where VAF*ᵢ* represents the variant allele frequency of variant *i* and *n* represents the number of selected variants in a given population.

Under Hardy–Weinberg equilibrium assumptions, the estimated carrier frequency was calculated as:

Carrier frequency = 2*Q*(1 − *Q*)

The modeled genetic prevalence of biallelic PORD was calculated as:

Modeled genetic prevalence = *Q*^2^

These estimates were interpreted as genetic prevalence rather than clinically observed prevalence or incidence. The calculation assumes random mating, accurate allele-frequency estimation, complete detection of relevant variants, and 100% penetrance of included disease-associated variant combinations. Because penetrance and expressivity may vary in PORD, and because predicted missense variants may not be truly disease-causing, estimates based on the expanded variant set were considered exploratory.

The estimated annual number of newborns with genetically predicted PORD in Mainland China and globally was calculated using the modeled genetic prevalence and published birth statistics from Mainland China and United Nations population data sources.

### Statistical analysis

Continuous variables were summarized as medians with ranges or interquartile ranges, as appropriate. Categorical variables were summarized as numbers and percentages using available-case denominators. Skeletal malformation scores between groups were compared using the Mann–Whitney U test for two-group comparisons or the Kruskal–Wallis test for multiple-group comparisons. Because the cohort was small, retrospective, and derived partly from heterogeneous published case reports, all genotype–phenotype analyses were considered exploratory. *P* values were interpreted descriptively, and no definitive causal inference was made from subgroup comparisons. Multiple testing was not formally adjusted; this limitation was stated in the section titled ‘Discussion’. Heatmaps of variant allele frequencies were generated using the pheatmap package in R (version 4.3.2). Graphs were plotted using GraphPad Prism version 8. A two-sided *P* value < 0.05 was considered nominally significant for exploratory comparisons.

## Results

### Case 1

A 15-year-old girl with congenital brachydactyly and a history of atypical external genitalia presented to the endocrinology department with an 11-month history of oligomenorrhea. She had undergone genital reconstructive surgery for partial labial fusion at approximately 2 years of age and laparoscopic bilateral ovarian cystectomy one month before presentation. Detailed neonatal genital findings, including clitoral size, number of perineal openings, and external genitalia score, were not available from the historical records.

**Figure 1:**
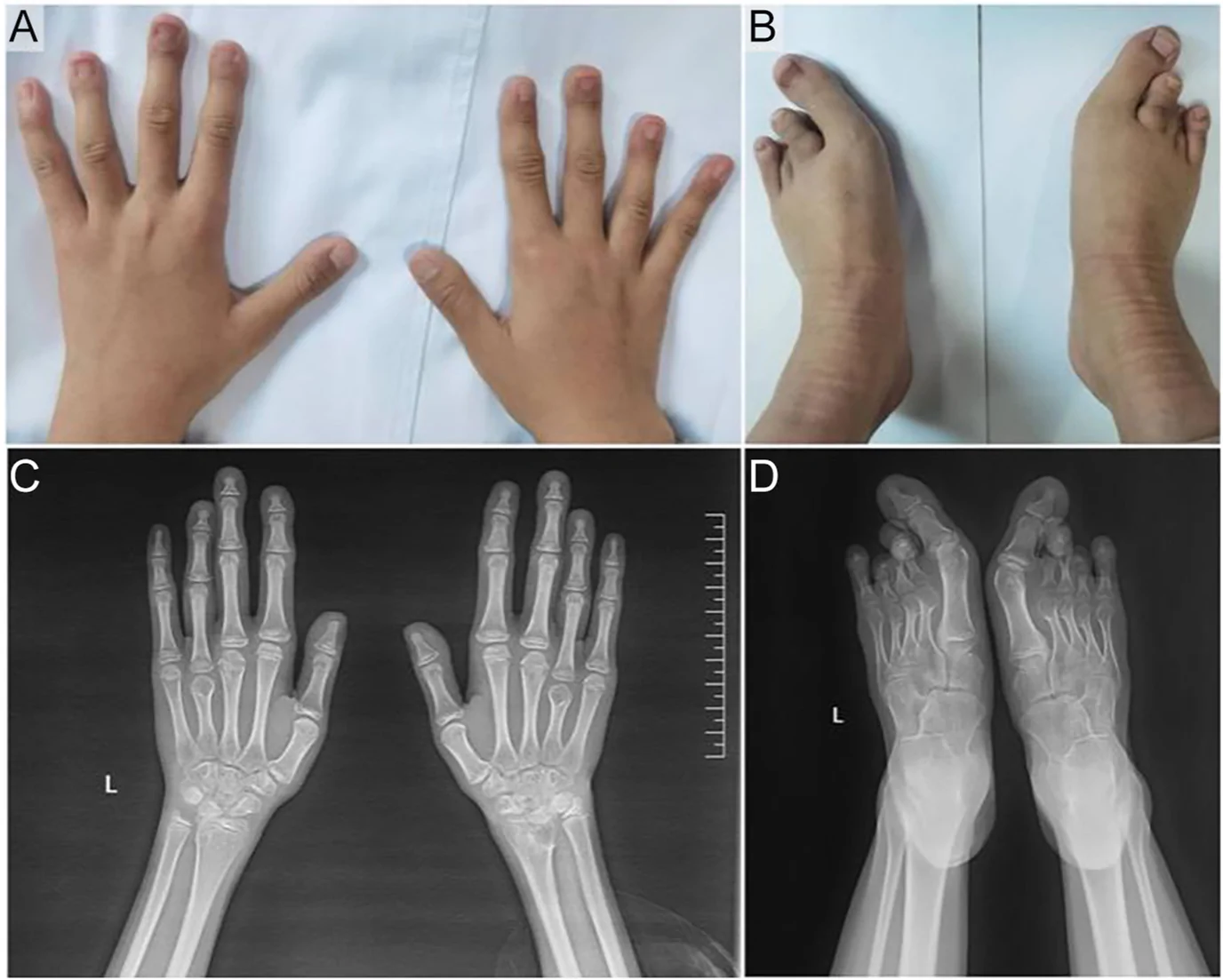
Clinical and radiographic skeletal findings in case 1. Clinical photographs showing shortening of the fingers (A) and toes (B). Radiographs confirmed shortening of the metacarpal bones of both hands (C) and metatarsal bones of both feet (D).

Physical examination revealed a broad nasal bridge and shortening of the fourth metacarpal bones of both hands and the second to fifth metatarsal bones of both feet. Radiography confirmed skeletal malformations of the hands and feet (Fig. 1). Pelvic ultrasonography showed a uterus and bilateral ovaries. Her karyotype was 46,XX. Abdominal imaging showed no adrenal mass or obvious adrenal enlargement. Blood pressure and serum electrolytes were within the reference ranges, and there was no clinical evidence of mineralocorticoid excess.

Laboratory testing showed a biochemical pattern compatible with impaired steroidogenesis, including increased serum follicle-stimulating hormone, 17-hydroxyprogesterone, and progesterone concentrations. Basal cortisol was not frankly deficient, but adrenal reserve was considered potentially impaired in view of the abnormal steroid precursor accumulation. ACTH stimulation testing was not performed before glucocorticoid treatment; therefore, pretreatment adrenal reserve could not be formally assessed. Serum sodium, potassium, blood pressure, renin, and aldosterone did not suggest clinically significant mineralocorticoid deficiency or excess. The coexistence of elevated progesterone and 17-hydroxyprogesterone with menstrual disturbance was consistent with the biochemical phenotype reported in non-classic or adolescent/adult presentations of PORD.

**Figure 2:**
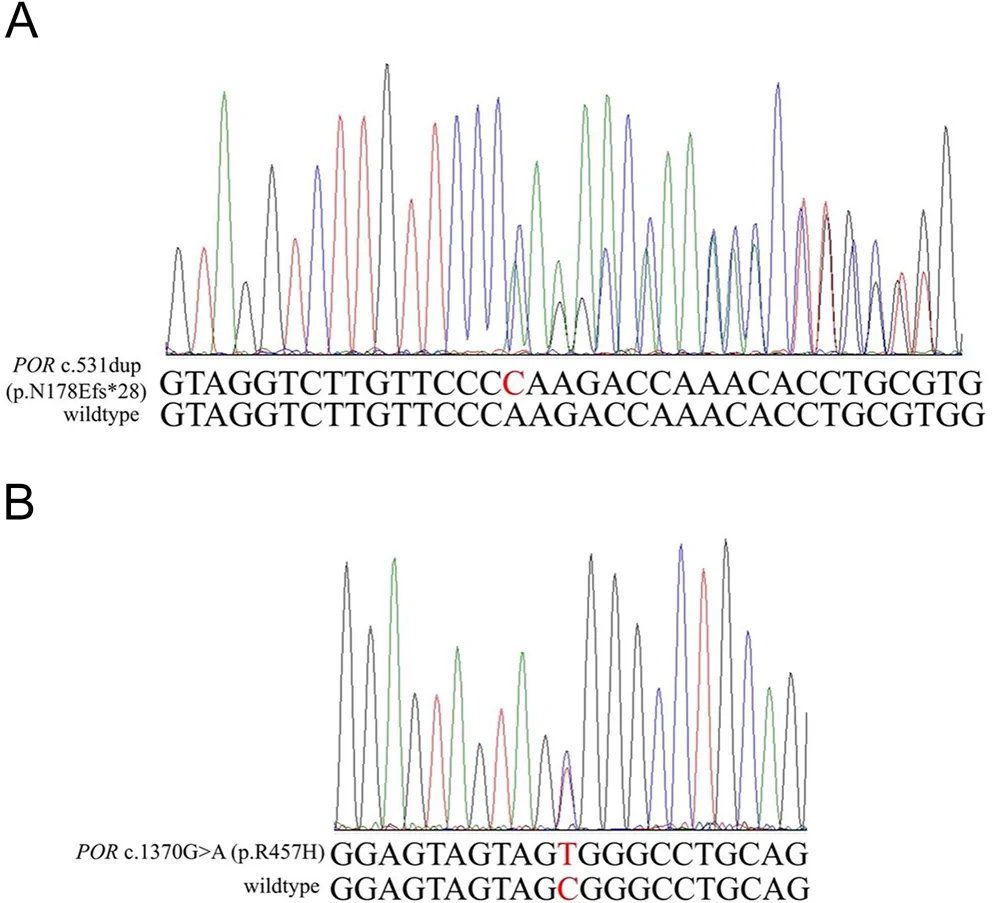
Sanger sequencing validation of POR variants in case 1. Sequencing chromatograms showing the novel frameshift variant c.531dup:p.N178Efs*28 and the recurrent missense variant c.1370G>A:p.R457H in POR in the proband.

Whole-genome sequencing identified compound heterozygous POR variants: c.1370G>A:p.R457H and the novel frameshift variant c.531dup:p.N178Efs*28 (Fig. 2). Sanger sequencing confirmed that the two variants were inherited in trans, supporting a molecular diagnosis of PORD (Supplementary Figs 4 and 5). The p.R457H variant has been recurrently reported in East Asian patients with PORD, whereas c.531dup:p.N178Efs*28 has not been previously reported in Chinese patients. The novel c.531dup:p.N178Efs*28 variant is predicted to introduce a premature termination codon and to result in nonsense-mediated mRNA decay or a severely truncated POR protein. According to ACMG/AMP criteria, this variant was classified as likely pathogenic, based on predicted loss of function, rarity or absence from population databases, detection in trans with the recurrent disease-associated p.R457H variant, and consistency between the patient’s phenotype and PORD.

The patient received glucocorticoid therapy with prednisone 5 mg/day. The treatment was initiated to suppress abnormal adrenal steroid precursor accumulation and improve menstrual irregularity rather than for documented adrenal crisis. At 6-month follow-up, her menstrual pattern had improved.

### Case 2

A 31-year-old woman was admitted to the Endocrinology Department of the First Affiliated Hospital of Zhengzhou University because of menstrual disorder. She had been diagnosed with hypothyroidism four years earlier and was receiving levothyroxine sodium tablets. Menarche occurred at 14 years of age. One year before presentation, she developed oligomenorrhea and subsequently received hormone replacement therapy. She had undergone two cycles of *in vitro* fertilization with frozen embryo transfer at a local hospital, without successful pregnancy.

Pelvic ultrasonography showed a right ovarian cyst. Physical examination was unremarkable, with no obvious skeletal malformation or external genital abnormality at adult presentation. Blood pressure and serum electrolytes were within the reference ranges, and there was no clinical evidence of mineralocorticoid excess or adrenal crisis. Laboratory testing showed increased serum 17-hydroxyprogesterone. The combination of menstrual disorder, infertility, ovarian cysts, and elevated 17-hydroxyprogesterone was compatible with a mild or non-classic adult presentation of PORD, which may overlap clinically with polycystic ovary syndrome or other reproductive endocrine disorders.

Whole-exome sequencing identified two POR variants: c.1370G>A:p.R457H and c.1820A>G:p.Y607C. The p.R457H variant is a recurrent East Asian-enriched POR variant, and p.Y607C has previously been reported in patients with PORD. Parental samples were not available; therefore, the phase of c.1370G>A:p.R457H and c.1820A>G:p.Y607C could not be experimentally confirmed. However, the patient’s endocrine and reproductive phenotype, together with the presence of two disease-associated POR variants, supported a clinical diagnosis of PORD. The clinical, biochemical, imaging, and genetic features of the two newly reported cases are summarized in Table 1.

**Table 1:** Clinical, biochemical, imaging, and genetic features of the two newly reported cases.

Feature	Case 1	Case 2
Age at presentation	15 years	31 years
Sex	Female	Female
Main presentation	Oligomenorrhea for 11 months	Menstrual disorder and infertility
Relevant medical history	Congenital brachydactyly; atypical external genitalia; genital reconstructive surgery for partial labial fusion at approximately 2 years of age; laparoscopic bilateral ovarian cystectomy 1 month before presentation	Hypothyroidism diagnosed 4 years earlier and treated with levothyroxine; oligomenorrhea; two unsuccessful cycles of *in vitro* fertilization and frozen embryo transfer
Menarche	Not available from historical record	14 years
Menstrual/reproductive phenotype	Oligomenorrhea; bilateral ovarian cysts	Oligomenorrhea; infertility; right ovarian cyst
Karyotype	46,XX	Not available
External genital phenotype	History of atypical external genitalia and partial labial fusion requiring surgery	No obvious external genital abnormality at adult presentation
Detailed neonatal genital findings	Not available; clitoral size, number of perineal openings, and external genitalia score were unavailable	Not applicable; no genital abnormality was reported at adult presentation
Internal genital imaging	Uterus and bilateral ovaries present	Right ovarian cyst detected by pelvic ultrasonography
Skeletal phenotype	Broad nasal bridge; shortened fourth metacarpal bones of both hands; shortened second to fifth metatarsal bones of both feet	No obvious skeletal malformation at adult presentation
Radiographic skeletal findings	Radiographs confirmed skeletal malformations of the hands and feet	Skeletal radiography was not clinically indicated because physical examination was unremarkable
Adrenal imaging	No adrenal mass or obvious adrenal enlargement	Not available
ACTH stimulation test	Not performed before glucocorticoid treatment	Not available
Adrenal reserve interpretation	Pretreatment adrenal reserve could not be formally assessed	Adrenal reserve could not be formally assessed from available data
17-Hydroxyprogesterone	48.2 nmol/L, increased	35.7 nmol/L, increased
Progesterone	46.6 nmol/L, increased	32.1 nmol/L, increased
Pregnenolone	14.1 ng/mL, increased/PORD-compatible	11.4 ng/mL, increased/PORD-compatible
Deoxycorticosterone	1.4 ng/mL, normal to mildly increased/PORD-compatible	1.1 ng/mL, normal to mildly increased/PORD-compatible
DHEA-S	187.7 μg/dL, variable/PORD-compatible	194.2 μg/dL, variable/PORD-compatible
Androstenedione	3.4 ng/mL, within or near upper female reference range	3.9 ng/mL, within or near upper female reference range
Testosterone	0.67 ng/mL, within or mildly above typical female reference range depending on laboratory cutoff	0.45 ng/mL, within typical female reference range
Estradiol	221 pg/mL, interpretation depends on menstrual phase	184 pg/mL, interpretation depends on menstrual phase
LH	14.8 IU/L, mildly increased or upper-range depending on menstrual phase	16.2 IU/L, mildly increased or upper-range depending on menstrual phase
FSH	28.1 IU/L, increased	18.3 IU/L, mildly increased or upper-range depending on menstrual phase
Main biochemical interpretation	Elevated 17-hydroxyprogesterone, progesterone, and pregnenolone with menstrual disturbance supported impaired steroidogenesis compatible with adolescent PORD	Elevated 17-hydroxyprogesterone, progesterone, and pregnenolone with infertility and ovarian cyst supported a mild/adult POR-associated reproductive endocrine phenotype
Genetic testing method	Whole-genome sequencing with Sanger validation	Whole-exome sequencing
POR variant 1	c.1370G>A:p.R457H	c.1370G>A:p.R457H
POR variant 2	c.531dup:p.N178Efs*28	c.1820A>G:p.Y607C
Variant phase	Confirmed in trans by parental Sanger sequencing	Not experimentally confirmed because parental samples were unavailable
Parental origin	c.531dup:p.N178Efs*28 inherited from father; c.1370G>A:p.R457H inherited from mother	Not available
Variant interpretation	p.R457H is recurrent in East Asian PORD; p.N178Efs*28 is a novel frameshift variant classified as likely pathogenic	p.R457H is recurrent in East Asian PORD; p.Y607C has previously been reported in patients with PORD
ACMG/AMP interpretation	c.531dup:p.N178Efs*28 classified as likely pathogenic based on predicted loss of function, rarity or absence in population databases, trans configuration with disease-associated p.R457H, and phenotype consistency	Formal ACMG/AMP interpretation limited by lack of phase confirmation
Treatment	Prednisone 5 mg/day	PORD-specific treatment not clearly documented
Treatment indication	Suppression of abnormal steroid precursor accumulation and improvement of menstrual irregularity; not treatment for documented adrenal crisis	Reproductive endocrine management
Follow-up	Menstrual pattern improved after 6 months	Follow-up outcome not available
Final case interpretation	Genetically confirmed PORD with skeletal malformations, 46,XX DSD history, ovarian cysts, menstrual disturbance, elevated steroid precursors, and biallelic POR variants confirmed in trans	Clinically supported POR-associated PORD phenotype with menstrual disorder, infertility, ovarian cyst, elevated steroid precursors, and two disease-associated POR variants of unconfirmed phase

Hormone interpretation was based on age-, sex-, menstrual-phase-, and assay-specific reference ranges from the testing laboratory. ACTH stimulation testing was not performed before glucocorticoid treatment in case 1. In case 2, the phase of the two POR variants could not be experimentally confirmed because parental samples were unavailable.

### Identification of published Chinese PORD cases

The literature search identified 42 records, including 34 Chinese-language records and 8 English-language records. After excluding 6 review articles and 1 duplicate publication, 35 articles reporting 50 putative Chinese PORD patients were assessed for eligibility. Twelve patients were excluded because of duplicate reporting, incomplete genotyping, presence of only one reported POR variant, insufficient clinical information, or variants considered too common in the Chinese population to support monogenic PORD. Finally, 38 literature-derived patients were included. Together with the two newly reported probands, the pooled cohort comprised 40 unique Chinese patients with PORD (Supplementary Fig. 1; Supplementary Tables S2 and S3) (11, 12, 13, 14, 15, 16, 17, 18, 19, 20, 21, 22, 23, 24, 25, 26, 27, 28, 29, 30, 31, 32, 33, 34, 35, 36, 37, 38). Among patients with available data, maternal virilization during pregnancy was reported in 13/19 patients (68.4%), adrenal insufficiency in 8/35 patients (22.9%), and DSD or atypical sex development in 29/36 patients (80.6%) (Supplementary Table S4).

### Clinical and genetic spectrum of Chinese patients with PORD

The first Chinese patient with PORD was reported in 2009 (11). No additional Chinese cases were identified until 2016, after which reports appeared almost every year, with the largest number of cases reported in 2019. Among the pooled 40 patients, 28 were female, and 12 were male. Seven patients were diagnosed at age 1 year or younger.

A total of 24 distinct POR variants were identified in the pooled Chinese cohort. Among 80 alleles, the most frequent variant was c.1370G>A:p.R457H, accounting for 40/80 alleles (50.0%). Other recurrent variants included c.1820A>G:p.Y607C (7/80, 8.8%), c.744C>G:p.Y248* (3/80, 3.8%), c.517-19_517-10delGGCCCCTGTGinsC (2/80, 2.5%), c.1195_1203del:p.P399_E401del (2/80, 2.5%), c.667C>T:p.R223* (2/80, 2.5%), c.976T>G:p.Y326D (2/80, 2.5%), and c.1660C>T:p.R554* (2/80, 2.5%). The remaining variants were singletons. These findings indicate that p.R457H is a recurrent East Asian-enriched POR variant in Chinese patients; however, founder-effect status should be interpreted cautiously unless supported by haplotype or population-genetic evidence.

### Skeletal manifestations and genotype groups

Patients were classified into four genotype groups: group A, R457H/R457H; group B, R457H/null; group C, R457H/other; and group D, other/null or other/other (Supplementary Fig. 2). Because skeletal findings were extracted retrospectively from published reports, unreported skeletal features were treated as missing rather than absent. Therefore, comparisons of skeletal malformation scores were considered exploratory.

**Figure 3:**
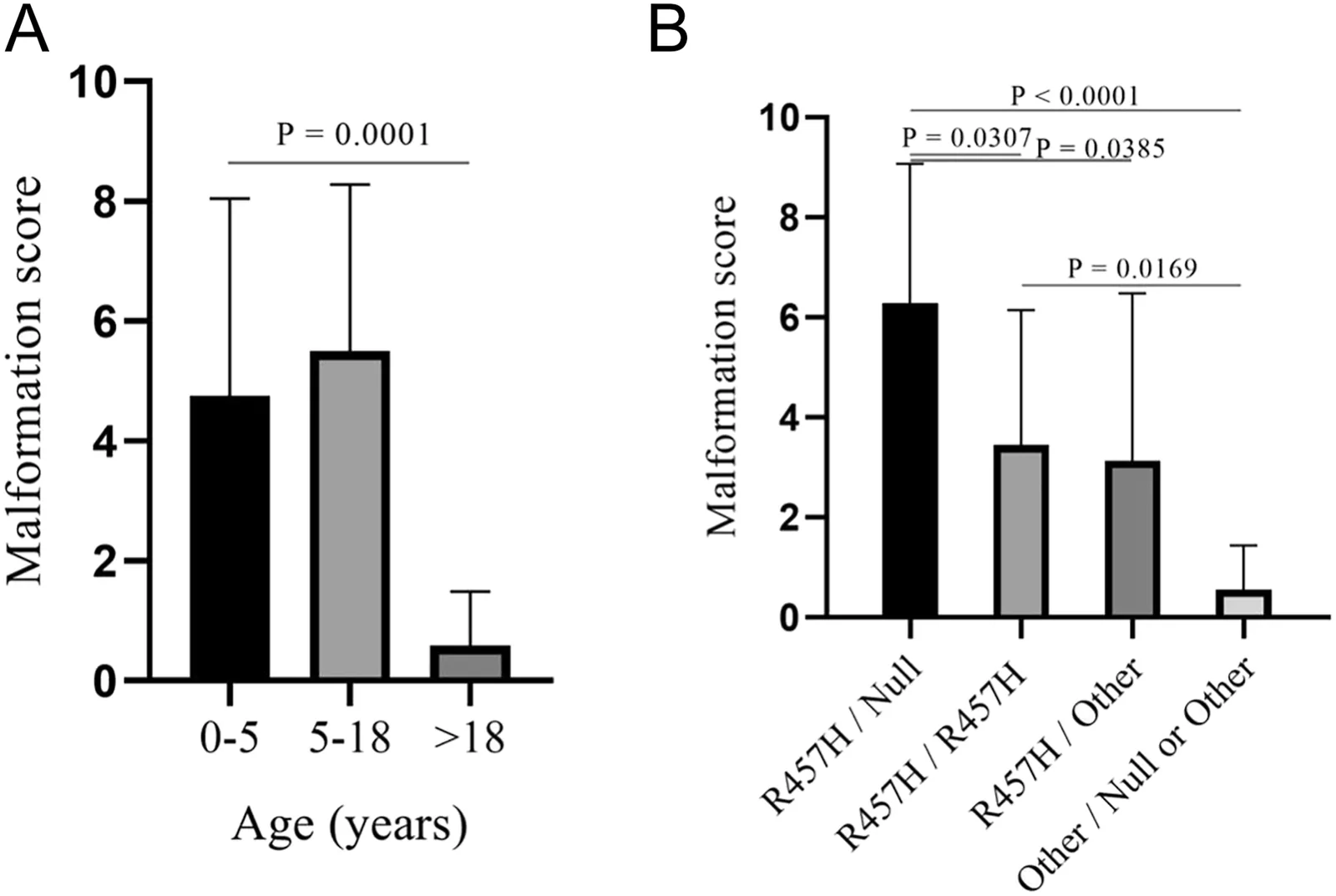
Skeletal malformation scores stratified by age and genotype. Comparison of skeletal malformation scores according to age group (A) and POR genotype group (B). Genotype groups were defined as R457H/R457H, R457H/null, R457H/other, and other/null or other/other.

All patients in group B had reported skeletal abnormalities. In contrast, 2/9 patients (22.2%) in group A, 3/8 patients (37.5%) in group C, and 6/9 patients (66.7%) in group D had a skeletal malformation score of 0. There was no significant difference in skeletal malformation scores between males and females (*P* = 0.2043). Skeletal malformation scores were higher in children than in adults (*P* = 0.0001; Fig. 3A).

Across genotype groups, patients with R457H/null variants had higher skeletal malformation scores than patients in group D (*P* < 0.0001), group C (*P* = 0.0385), and group A (*P* = 0.0307; Fig. 3B). Patients with R457H/R457H also had higher skeletal malformation scores than patients in group D (*P* = 0.0169). Because of the small subgroup sizes, retrospective data extraction, missing skeletal assessments, and multiple exploratory comparisons, these *P* values should be interpreted descriptively rather than as definitive genotype–phenotype associations.

### Maternal virilization, adrenal insufficiency, and DSD phenotypes

Maternal virilization during pregnancy was reported in 13 of 19 mothers with available pregnancy information (68.4%). Maternal virilization was more frequently reported in pregnancies of patients carrying p.R457H, particularly in the R457H/null genotype group. However, because pregnancy history was unavailable for several patients and the subgroup sizes were small, this genotype-associated pattern was interpreted descriptively rather than as a definitive genotype–phenotype association.

Adrenal insufficiency data were available for 35 patients. Adrenal insufficiency was reported in 8 of 35 patients (22.9%). No clear difference in reported adrenal insufficiency was observed among the genotype groups. However, adrenal function was assessed heterogeneously across the included reports, and ACTH stimulation testing was not consistently performed or reported. Therefore, the frequency of adrenal insufficiency should be interpreted cautiously and may not reflect the true prevalence of impaired adrenal reserve in Chinese patients with PORD.

DSD or atypical sex-development phenotypes were reported in 29 of 36 patients with available data (80.6%). These phenotypes occurred across multiple genotype groups and were not restricted to a single POR variant combination. Reported manifestations included 46,XX virilization, atypical external genitalia, labial fusion, clitoromegaly, history of genital surgery, and 46,XY undervirilization where karyotype information was available. However, the severity of genital atypia was not uniformly described in the published reports, and external genitalia score or Prader stage was rarely available. Therefore, DSD frequency was calculated using available-case denominators, and missing genital descriptions were treated as unavailable rather than normal.

### Variants identified in population databases

A total of 1,595 POR variants were identified in gnomAD, and 4,040 POR variants were identified in WBBC (Supplementary Table S5). The larger number of variants identified in WBBC, despite its smaller sample size, likely reflects differences in sequencing strategy, variant discovery approach, population-specific variant representation, and the greater ability of whole-genome sequencing to capture non-coding and intronic variants. Therefore, direct comparison of raw variant counts between gnomAD and WBBC should be interpreted cautiously.

**Figure 4:**
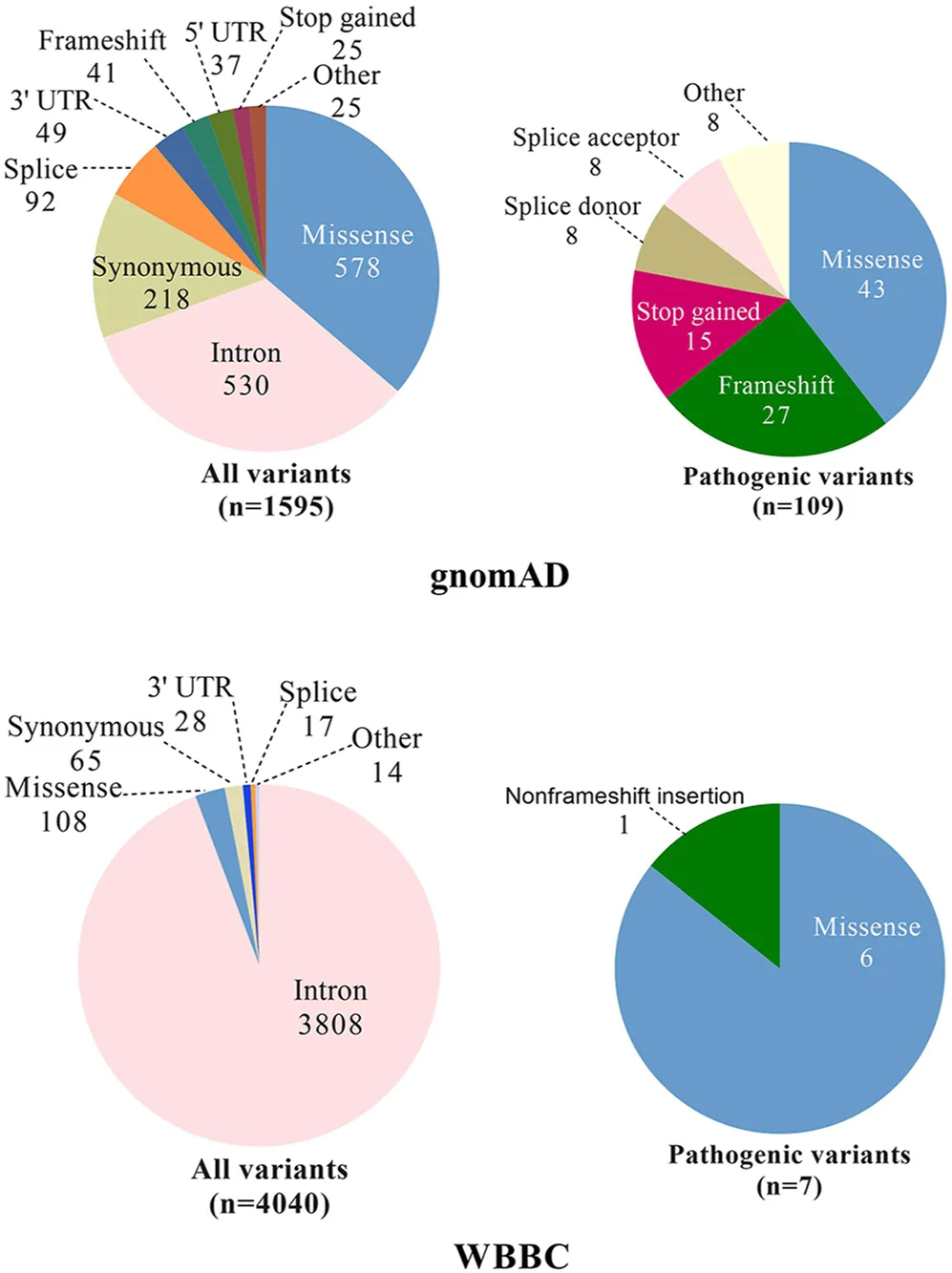
Distribution of POR variant classes in gnomAD and WBBC. Pie charts showing the distribution of variant classes among all identified POR variants (left panels) and among candidate deleterious POR variants included in the expanded exploratory set (right panels). gnomAD, Genome Aggregation Database; WBBC, Westlake BioBank for Chinese; UTR, untranslated region.

In gnomAD, the most common classes of all POR variants were missense variants (*n* = 578, 36.2%), intronic variants (*n* = 530, 33.2%), synonymous variants (*n* = 218, 13.7%), and splice-site variants (*n* = 92, 5.8%) (Fig. 4). In WBBC, most identified POR variants were intronic (*n* = 3,808, 94.3%), followed by missense (*n* = 108, 2.7%), synonymous (*n* = 65, 1.6%), and 3′ untranslated region (*n* = 28, 0.7%).

After variant filtering and classification, variants were grouped into conservative, moderate, and expanded exploratory sets as described in the section titled ‘Methods’. The expanded exploratory set contained 116 candidate deleterious POR variants, including 109 variants in gnomAD and 7 variants in WBBC (Supplementary Fig. 3; Supplementary Table S1). These variants were not all classified as pathogenic by ACMG/AMP criteria. Therefore, they are referred to as candidate deleterious variants rather than pathogenic variants throughout the population-genetic analysis.

In the expanded exploratory gnomAD set, missense variants (*n* = 43, 39.4%), frameshift variants (*n* = 27, 24.8%), and stop-gain variants (*n* = 15, 13.8%) were overrepresented. In WBBC, 6 of 7 candidate deleterious variants (85.7%) were missense variants. In gnomAD, 48 variants (44.0%) had a global variant allele frequency below 0.001%, and 54 variants (49.5%) were singletons. The highest global allele frequency in gnomAD was observed for p.A287P (rs121912974; VAF = 0.024%). In WBBC, the allele frequencies of the seven candidate deleterious variants ranged from 0.01 to 0.05%; p.R457H (rs28931608) and p.R550Q had the highest allele frequencies (0.045%).

### Population distribution of candidate deleterious POR variants

In gnomAD, the largest number of candidate deleterious POR variants in the expanded exploratory set was observed in non-Finnish Europeans (*n* = 66), followed by African/African American individuals (*n* = 27), Latino/Admixed American individuals (*n* = 23), South Asians (*n* = 22), East Asians (*n* = 17), Finnish individuals (*n* = 10), other/unspecified ancestry individuals (*n* = 8), and Ashkenazi Jewish individuals (*n* = 1).

The highest variant allele frequency for a candidate deleterious POR variant was observed in South Asians for p.Y190C (rs782681272; VAF = 0.16%). This corresponded to approximately 32.4 carriers per 10,000 individuals for this variant in South Asians, more than 50-fold higher than in non-Finnish Europeans (0.64 per 10,000 individuals). Ten South Asian-specific candidate deleterious variants were observed, six of which were singletons.

In African/African American individuals, 13 candidate deleterious variants were ancestry-specific in the gnomAD dataset. The highest allele frequency among African/African American-specific variants was observed for p.D641Y (rs782153040; VAF = 0.013%), whereas the remaining 12 variants were singletons. The highest allele frequency in African/African American individuals was observed for p.R371C (rs782193817; VAF = 0.029%), corresponding to approximately 5.8 carriers per 10,000 individuals.

In Latino/Admixed American individuals, seven ancestry-specific candidate deleterious variants were observed. The highest allele frequency was found for p.R202P (rs547249232; VAF = 0.032%), corresponding to approximately 6.4 carriers per 10,000 individuals. In Ashkenazi Jewish individuals, only one candidate deleterious variant, p.R673H (rs376693603), was observed (VAF = 0.02%).

In East Asians, six ancestry-specific candidate deleterious variants were observed, all of which were singletons. The highest allele frequency in East Asians was observed for p.R457H (VAF = 0.056%), corresponding to approximately 11.3 carriers per 10,000 individuals. This variant was absent from African/African American, Latino/Admixed American, Ashkenazi Jewish, Finnish, and South Asian populations in gnomAD and was also observed in WBBC. These findings support enrichment of p.R457H in East Asian and Chinese populations. Because haplotype analysis was not performed, the data support an East Asian-enriched recurrent variant but do not by themselves prove a founder effect.

In Finnish individuals, two ancestry-specific frameshift variants were observed, p.L564DfsTer12 and p.E56SfsTer8, each with a VAF of 0.029%. The highest Finnish allele frequency was observed for p.V472AfsTer102 (VAF = 0.057%), corresponding to approximately 11.5 carriers per 10,000 individuals. In non-Finnish Europeans, 39 ancestry-specific candidate deleterious variants were observed, 26 of which were singletons. The highest allele frequency in non-Finnish Europeans was observed for p.A287P (rs121912974; VAF = 0.037%). No ancestry-specific candidate deleterious variant was found in the other/unspecified ancestry group; the highest allele frequency in this group was observed for p.R522H (VAF = 0.092%). These findings are shown in Supplementary Fig. 6.

### Carrier frequency and modeled genetic prevalence of PORD

In the conservative analysis, the modeled genetic prevalence was 0.78 per 1,000,000 in the gnomAD global population, 0.51 per 1,000,000 in gnomAD East Asians, and 0.31 per 1,000,000 in WBBC Chinese individuals (Table 2). In the moderate variant set, the corresponding estimates were 2.05, 1.13, and 0.31 per 1,000,000, respectively. Using the expanded exploratory set, the estimates increased to 7.76, 3.27, and 2.44 per 1,000,000, respectively, indicating that prevalence estimates were sensitive to variant-classification stringency (Table 2).

**Table 2:** Carrier frequency and modeled genetic prevalence by variant set.

Variant set	Population	Carrier frequency	Modeled prevalence per 1,000,000
Set 1 conservative	gnomAD global	0.00176	0.78
Set 1 conservative	gnomAD East Asian	0.00143	0.51
Set 1 conservative	WBBC Chinese	0.00112	0.31
Set 2 moderate	gnomAD global	0.00286	2.05
Set 2 moderate	gnomAD East Asian	0.00213	1.13
Set 2 moderate	WBBC Chinese	0.00112	0.31
Set 3 expanded exploratory	gnomAD global	0.00556	7.76
Set 3 expanded exploratory	gnomAD East Asian	0.00361	3.27
Set 3 expanded exploratory	WBBC Chinese	0.00312	2.44

In gnomAD ancestry groups, the highest estimated carrier frequency and modeled genetic prevalence were observed in South Asians (carrier frequency 0.00828; modeled genetic prevalence 17.3 per 1,000,000), followed by non-Finnish Europeans (0.00549; 7.6 per 1,000,000), other/unspecified ancestry individuals (0.00527; 7.0 per 1,000,000), African/African American individuals (0.00526; 6.9 per 1,000,000), Finnish individuals (0.00424; 4.5 per 1,000,000), Latino/Admixed American individuals (0.00389; 3.8 per 1,000,000), East Asians (0.00361; 3.3 per 1,000,000), and Ashkenazi Jewish individuals (0.000392; 0.038 per 1,000,000).

These estimates represent modeled genetic prevalence rather than clinically observed prevalence or incidence. Because the expanded exploratory variant set included predicted deleterious variants that have not all been confirmed as pathogenic or likely pathogenic by ACMG/AMP criteria, the estimates should be interpreted cautiously and regarded as upper-bound exploratory estimates. The results also assume Hardy–Weinberg equilibrium, random mating, complete penetrance, and accurate detection and classification of disease-associated POR variants.

Based on the 2022 birth statistics for Mainland China, the WBBC-based modeled genetic prevalence predicted approximately 22.9 newborns with genetically predicted PORD per year in Mainland China. Using global birth statistics, the gnomAD-based model predicted approximately 1,045 newborns with genetically predicted PORD per year worldwide. These values should be interpreted as genetically modeled birth estimates, not as observed annual clinical incidence.

## Discussion

PORD is a rare form of congenital adrenal hyperplasia, but its clinical presentation differs substantially from classic CAH caused by single-enzyme defects such as 21-hydroxylase deficiency or 17α-hydroxylase deficiency (44). Because POR provides electrons to several microsomal cytochrome P450 enzymes, PORD can affect multiple steroidogenic and non-steroidogenic pathways simultaneously. Therefore, patients may present with a broad combination of adrenal steroid abnormalities, DSD, maternal virilization during pregnancy, skeletal malformations, menstrual disturbance, ovarian cysts, infertility, and variable adrenal insufficiency. Skeletal deformity is an important clue for distinguishing PORD from other forms of CAH, especially in patients with Antley–Bixler-like features. However, absence of skeletal abnormalities does not exclude PORD, particularly in adolescent or adult patients with milder reproductive endocrine presentations.

In the present study, we report two additional Chinese patients with PORD and systematically summarized published Chinese cases. The two newly reported cases illustrate the clinical heterogeneity of PORD. Case 1 presented in adolescence with congenital skeletal abnormalities, a history of atypical external genitalia, oligomenorrhea, ovarian cysts, and elevated steroid precursors. Case 2 presented in adulthood mainly with menstrual disorder, infertility, ovarian cysts, and elevated 17-hydroxyprogesterone, without obvious skeletal malformation at adult examination. These findings support the view that PORD should be considered not only in neonates or children with DSD and skeletal malformations but also in adolescent or adult women with unexplained menstrual disturbance, ovarian cysts, infertility, or biochemical features overlapping with non-classic CAH or polycystic ovary syndrome. Our pooled Chinese cohort showed that p.R457H was the most frequent POR variant, accounting for 40 of 80 alleles. This finding is consistent with previous reports that p.R457H is enriched in East Asian patients with PORD, whereas other variants, such as p.A287P, are more frequently reported in European-ancestry populations. However, although the high frequency of p.R457H in Chinese patients and its enrichment in East Asian population databases are compatible with a possible founder effect, our study did not perform haplotype analysis. Therefore, p.R457H should be described as an East Asian-enriched recurrent variant rather than definitively as a founder mutation.

The clinical review also showed frequent maternal virilization, DSD, and variable adrenal insufficiency. Maternal virilization was reported in 13 of 19 pregnancies with available information, and DSD or atypical sex-development phenotypes were reported in 29 of 36 patients with available data. These findings are biologically plausible because impaired placental and fetal steroid metabolism in PORD can alter androgen and estrogen balance. However, the severity of DSD was not uniformly reported in the literature-derived cases. External genitalia scores, Prader stages, detailed genital anatomy, and neonatal endocrine data were often unavailable. Therefore, the true frequency and severity distribution of DSD in Chinese patients with PORD remain uncertain. Future reports should describe genital phenotype using standardized terminology and scoring systems where possible. Adrenal insufficiency was reported in 8 of 35 patients with available adrenal-function information. This proportion should be interpreted cautiously because adrenal reserve was assessed heterogeneously across reports, and ACTH stimulation testing was not consistently performed. Basal cortisol alone may not be sufficient to exclude impaired adrenal reserve in PORD. From a management perspective, patients with suspected or confirmed PORD should undergo formal adrenal evaluation, including ACTH stimulation testing when clinically appropriate, and should receive stress-dose education if adrenal reserve is impaired. The genotype–phenotype analysis suggested that patients with R457H/null genotypes had more severe skeletal malformation scores than some other genotype groups. This observation is clinically interesting, but it should be interpreted as exploratory. The skeletal score was derived from retrospective case reports, missing skeletal features were common, and the score has not been independently validated in a Chinese PORD cohort. In addition, subgroup sizes were small, and multiple comparisons were performed. Therefore, these data suggest a possible genotype–skeletal phenotype relationship but do not establish a definitive association.

Compared with 21-OHD, PORD appears to be much rarer. 21-OHD, the most common form of CAH, is caused by pathogenic variants in CYP21A2. Neonatal screening studies have estimated the incidence of classic 21-OHD to be approximately 1 in 14,000 to 1 in 18,000 live births, with a heterozygous carrier frequency of approximately 1 in 60 (45). Based on gnomAD and Hardy–Weinberg equilibrium modeling, Xiao *et al.* reported a genetic prevalence of 21-OHD of 1.1 cases per 10,000 individuals globally and 9.6 cases per 10,000 individuals in East Asians (10). In contrast, using the expanded exploratory POR variant set, our study estimated a modeled genetic prevalence of PORD of 7.8 per 1,000,000 individuals globally, 3.3 per 1,000,000 individuals in East Asians, and 2.4 per 1,000,000 individuals in WBBC Chinese. These values are orders of magnitude lower than estimates for 21-OHD and are consistent with the much smaller number of clinically reported PORD cases. However, these are modeled genetic prevalence estimates, not clinically observed incidence rates.

A major strength of this study is the integration of clinical cases, a systematic review of Chinese patients, and population-database modeling. However, the population-genetic analysis has important limitations. First, variant classification strongly affects prevalence estimates. ClinVar-classified pathogenic or likely pathogenic variants and previously reported disease-causing variants provide stronger evidence than rare missense variants predicted to be damaging by *in silico* tools. Computational prediction alone cannot replace ACMG/AMP classification, segregation analysis, functional POR activity assays, or repeated observation in affected individuals. Therefore, estimates based on the expanded exploratory variant set may overestimate the true disease-causing allele burden.

Second, the Hardy–Weinberg-based approach assumes random mating, accurate allele-frequency estimation, complete detection of relevant variants, and complete penetrance of included disease-associated genotypes. These assumptions may not fully apply to PORD. PORD shows variable expressivity, and mild or non-classic cases may be underdiagnosed. Conversely, not all predicted deleterious variants necessarily cause clinically meaningful POR deficiency. Therefore, the calculated values should be interpreted as modeled genetic prevalence under defined assumptions rather than as observed clinical prevalence or incidence.

Third, gnomAD and WBBC are not directly equivalent datasets. gnomAD includes both exome and genome data from multiple ancestry groups, whereas WBBC provides Chinese population-specific data with whole-genome sequencing and high-density genotyping components. Differences in sample size, sequencing strategy, coverage, variant-calling pipeline, and ancestry structure may influence the number and type of POR variants detected. The larger number of total POR variants in WBBC despite its smaller sample size likely reflects, at least in part, differences in whole-genome variant discovery and detection of intronic variants. Therefore, cross-database comparisons should be interpreted cautiously.

Our findings have implications for genetic diagnosis. The high frequency of p.R457H among Chinese patients suggests that this variant is an important diagnostic clue in East Asian patients with suspected PORD. However, p.R457H-targeted testing alone is not sufficient as a general diagnostic strategy. Because PORD is genetically heterogeneous and its clinical presentation is often not pathognomonic, targeted testing for p.R457H may be useful only in selected patients with strong clinical suspicion or as part of an initial low-cost screen. In most patients with atypical CAH, DSD, skeletal malformations, infertility, or unexplained steroid abnormalities, broader approaches such as steroid profiling combined with CAH/DSD gene panels, exome sequencing, or genome sequencing are more appropriate.

The novel c.531dup:p.N178Efs*28 variant expands the known mutational spectrum of POR in Chinese patients. As a frameshift variant predicted to introduce a premature termination codon, it is expected to cause loss of function through nonsense-mediated mRNA decay or production of a severely truncated protein. Its detection in trans with the recurrent disease-associated p.R457H variant, together with the patient’s compatible clinical and biochemical phenotype, supports its classification as likely pathogenic according to ACMG/AMP criteria. Nevertheless, functional studies measuring POR-dependent enzyme activity would provide stronger evidence for its pathogenicity.

Several limitations should be acknowledged. First, the two newly reported cases were described retrospectively, and some clinically important data, including detailed neonatal genital anatomy, standardized external genitalia scoring, and complete adrenal reserve testing, were unavailable. In case 1, ACTH stimulation testing was not performed before glucocorticoid treatment; therefore, the presence and severity of adrenal insufficiency could not be definitively established. In case 2, the phase of the identified POR variants was not confirmed by parental or segregation testing; therefore, it remains unverified whether the variants were located in trans. These limitations warrant cautious interpretation of the clinical and genetic findings in the two cases. Second, the literature-derived cohort was based on case reports and case series, which are prone to publication bias. Severe pediatric cases, patients with skeletal malformations, and patients with unusual reproductive presentations are more likely to be reported than mild or asymptomatic cases. Third, published reports varied substantially in clinical detail, biochemical testing, imaging, genetic testing methods, and follow-up information. Fourth, the skeletal malformation score was applied retrospectively and should be considered exploratory. Fifth, the population-database estimates depend on variant classification and should not be interpreted as observed disease incidence.

Despite these limitations, this study provides the most comprehensive summary to date of the clinical and genetic spectrum of Chinese patients with PORD and highlights several clinically relevant points: p.R457H is highly recurrent in Chinese patients; PORD can present across the lifespan; adult women may present mainly with menstrual disturbance, ovarian cysts, or infertility; adrenal reserve requires formal assessment; and population-database modeling can provide useful but assumption-dependent estimates of genetic prevalence.

In conclusion, this study identified a heterogeneous clinical and genetic spectrum of PORD in Chinese patients. We reported two Chinese probands, including one carrying the novel POR frameshift variant c.531dup:p.N178Efs*28, and summarized 40 unique Chinese patients with PORD. p.R457H was the most frequent variant in the Chinese cohort and was enriched in East Asian population data, supporting its importance as a recurrent East Asian-associated POR variant. Using gnomAD and WBBC, we estimated carrier frequency and modeled genetic prevalence of PORD across populations. These estimates should be interpreted as genetic modeling results rather than observed clinical incidence, especially when predicted deleterious variants are included. Our findings provide a reference for clinical recognition, genetic diagnosis, and future studies of PORD in Chinese and East Asian populations.

## Supplementary materials























## Declaration of interest

The authors declare that there is no conflict of interest that could be perceived as prejudicing the impartiality of the work reported.

## Funding

This work was supported by the Natural Science Foundation of Henan Province (Grant No. 212300410246) and the Joint Construction Project of Henan Provincial Health Commission (LHGJ20230284).

## Author contribution statement

TW conceptualized the study and wrote the original draft of the manuscript. CL and CH designed the methodology. YZ and GQ conducted the formal analysis. LZ led the investigation and wrote the manuscript (review and editing). TW and CL curated the data. All authors have read and agreed to the published version of the manuscript.

## Ethics statement

All procedures performed in this study involving human participants were in accordance with the ethical standards of the institutional and/or national research committee and the 1964 Helsinki Declaration and its later amendments or comparable ethical standards. All subjects were approved by the First Affiliated Hospital of Zhengzhou University (2024-KY-0282-002).

## Data availability

Data are available from the corresponding author on request.
