# Supplementary material for: Clinical and genetic spectrum of cytochrome P450 oxidoreductase deficiency in Chinese patients
Source: Endocr Connect. 2026 Sep 8;15(9):e260247. doi: 10.1530/EC-26-0247 (PMC13588290; doi:10.1530/EC-26-0247)
Supplement: Supplementary file 1 [file EC-26-0247_supplementary_figure_1.pdf]

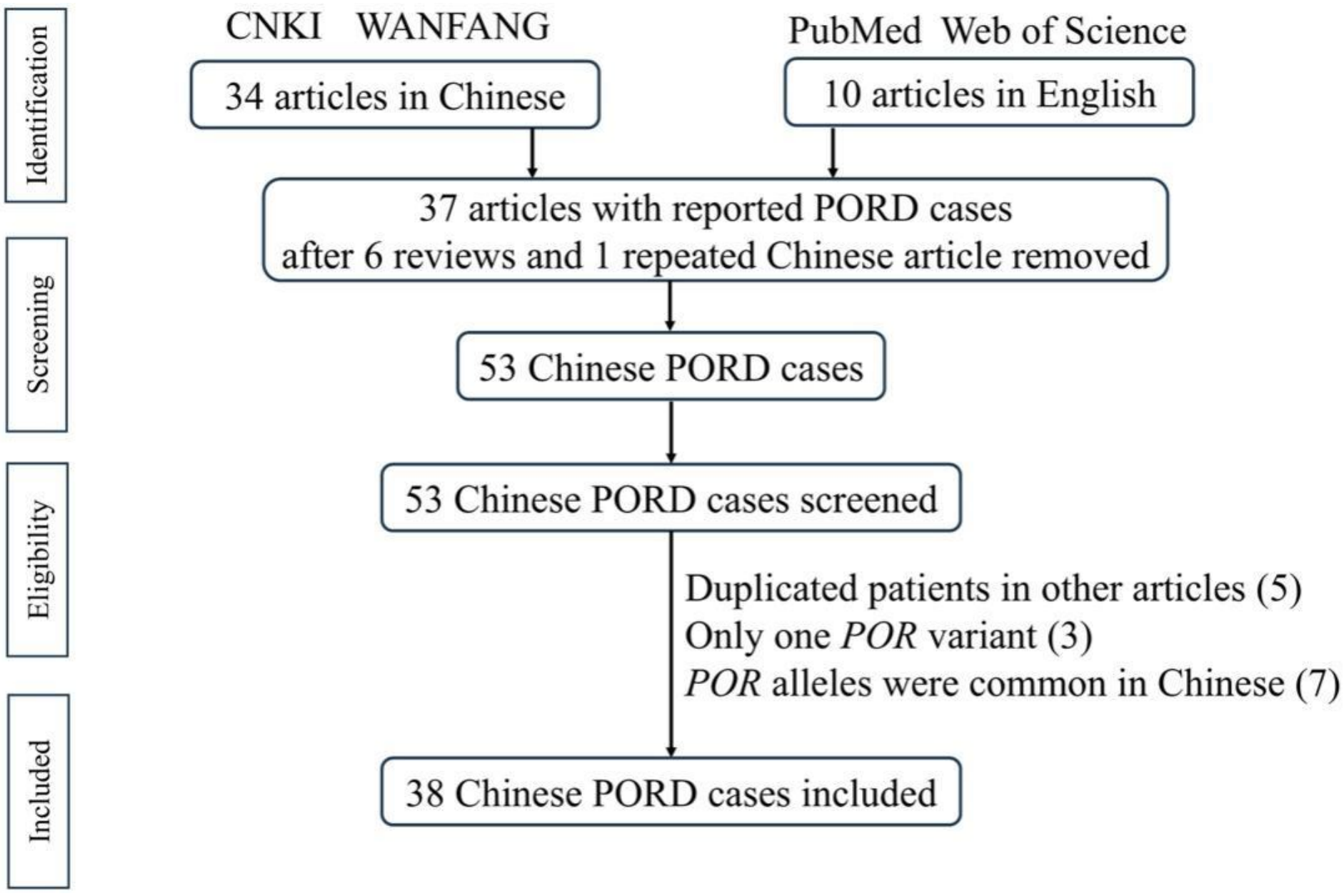
Identification
Screening
Eligibility
Included
CNKI WANFANG
34 articles in Chinese
PubMed Web of Science
10 articles in English
37 articles with reported PORD cases
after 6 reviews and 1 repeated Chinese article removed
53 Chinese PORD cases
53 Chinese PORD cases screened
Duplicated patients in other articles (5)
Only one POR variant (3)
POR alleles were common in Chinese (7)
38 Chinese PORD cases included
