# Supplementary material for: Clinical and genetic spectrum of cytochrome P450 oxidoreductase deficiency in Chinese patients
Source: Endocr Connect. 2026 Sep 8;15(9):e260247. doi: 10.1530/EC-26-0247 (PMC13588290; doi:10.1530/EC-26-0247)
Supplement: Supplementary file 2 [file EC-26-0247_supplementary_figure_2.pdf]

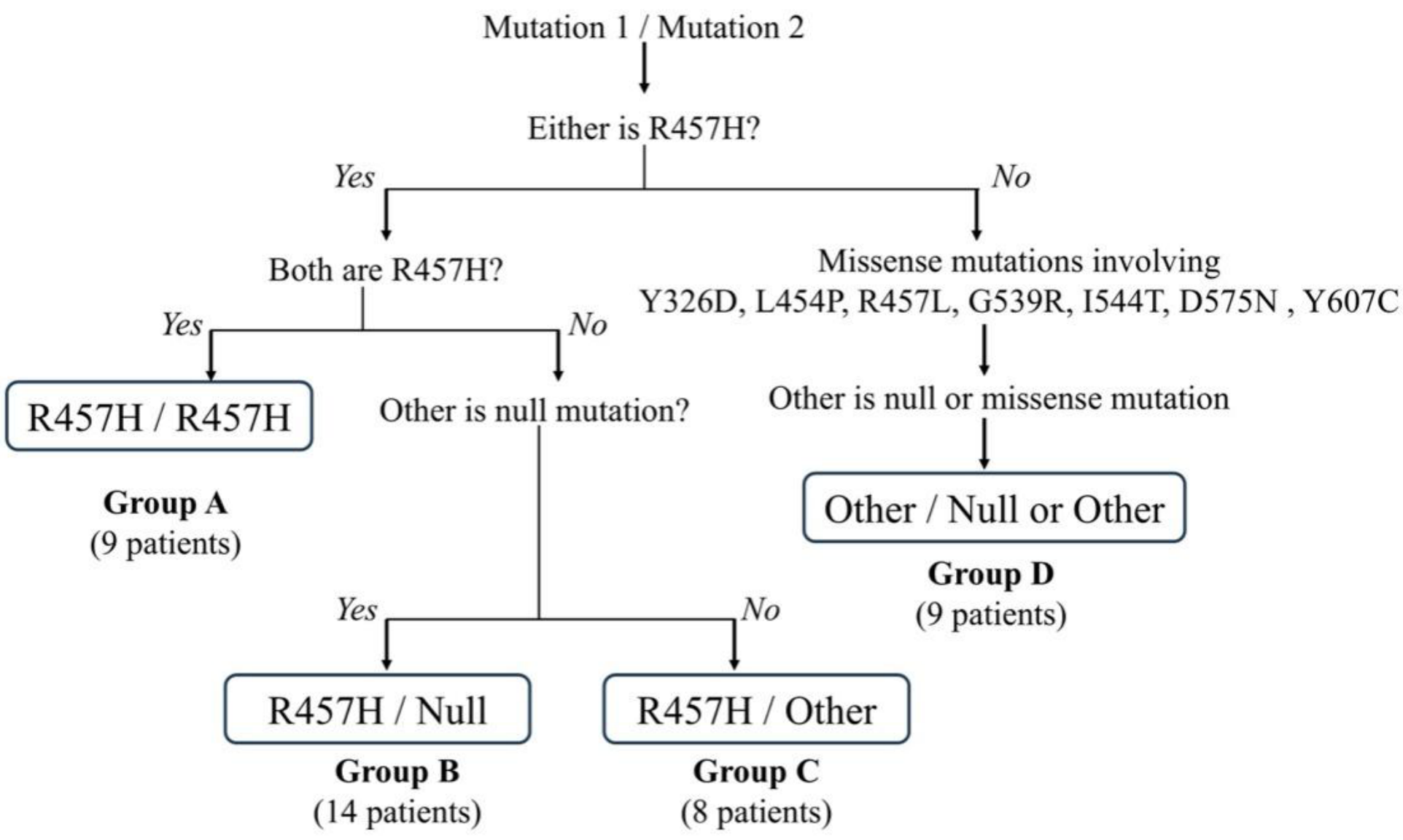

Mutation 1 / Mutation 2
Either is R457H?
Yes
No
Both are R457H?
Missense mutations involving
Y326D, L454P, R457L, G539R, I544T, D575N , Y607C
Yes
No
R457H / R457H
Other is null mutation?
Other is null or missense mutation
Other / Null or Other
Group A
(9 patients)
Group D
(9 patients)
Yes
No
R457H / Null
R457H / Other
Group B
(14 patients)
Group C
(8 patients)
