# Supplementary material for: Clinical and genetic spectrum of cytochrome P450 oxidoreductase deficiency in Chinese patients
Source: Endocr Connect. 2026 Sep 8;15(9):e260247. doi: 10.1530/EC-26-0247 (PMC13588290; doi:10.1530/EC-26-0247)
Supplement: Supplementary file 3 [file EC-26-0247_supplementary_figure_3.pdf]

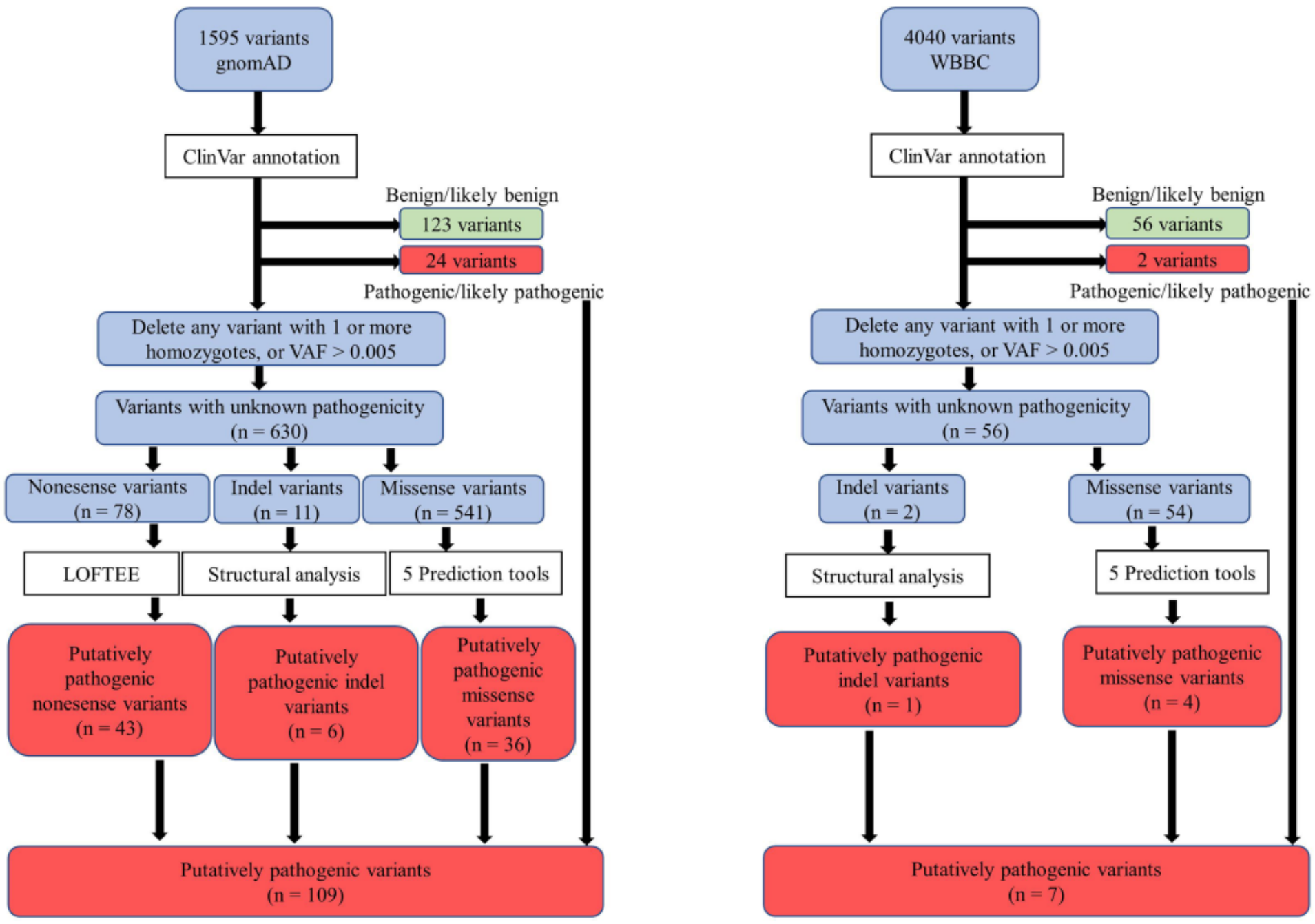
1595 variants gnomAD
ClinVar annotation
Benign/likely benign
123 variants
24 variants
Pathogenic/likely pathogenic
Delete any variant with 1 or more homozygotes, or VAF > 0.005
Variants with unknown pathogenicity (n = 630)
Nonsense variants (n = 78)
Indel variants (n = 11)
Missense variants (n = 541)
LOFTEE
Structural analysis
5 Prediction tools
Putatively pathogenic nonsense variants (n = 43)
Putatively pathogenic indel variants (n = 6)
Putatively pathogenic missense variants (n = 36)
Putatively pathogenic variants (n = 109)
4040 variants WBBC
ClinVar annotation
Benign/likely benign
56 variants
2 variants
Pathogenic/likely pathogenic
Delete any variant with 1 or more homozygotes, or VAF > 0.005
Variants with unknown pathogenicity (n = 56)
Indel variants (n = 2)
Missense variants (n = 54)
Structural analysis
5 Prediction tools
Putatively pathogenic indel variants (n = 1)
Putatively pathogenic missense variants (n = 4)
Putatively pathogenic variants (n = 7)
