# Supplementary material for: Clinical and genetic spectrum of cytochrome P450 oxidoreductase deficiency in Chinese patients
Source: Endocr Connect. 2026 Sep 8;15(9):e260247. doi: 10.1530/EC-26-0247 (PMC13588290; doi:10.1530/EC-26-0247)
Supplement: Supplementary file 4 [file EC-26-0247_supplementary_figure_4.pdf]

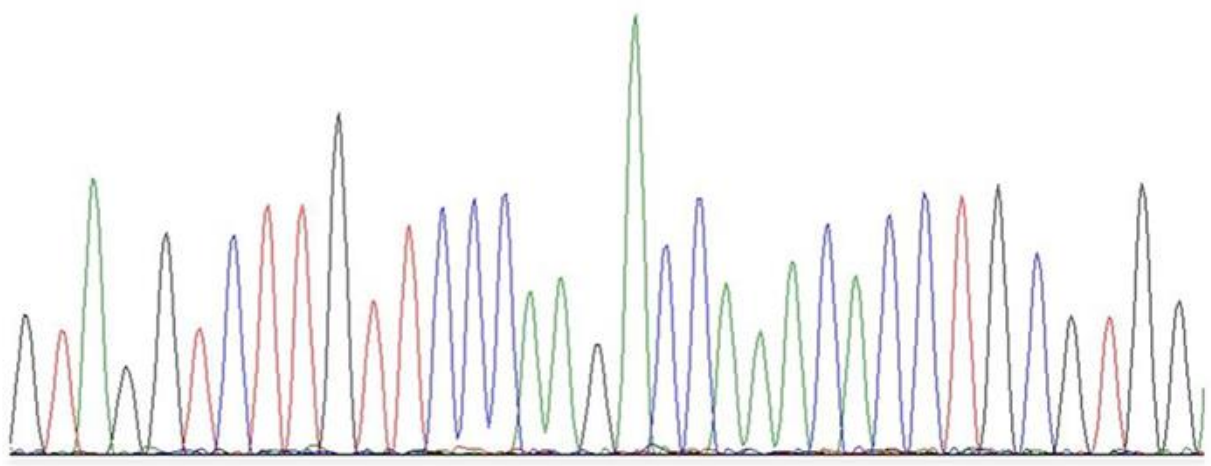

POR c.531dup
(p.N178Efs*28)

GTAGGTCTTGTTCCCAAGACCAAACACCTGCGTGG
