# Supplementary material for: Clinical and genetic spectrum of cytochrome P450 oxidoreductase deficiency in Chinese patients
Source: Endocr Connect. 2026 Sep 8;15(9):e260247. doi: 10.1530/EC-26-0247 (PMC13588290; doi:10.1530/EC-26-0247)
Supplement: Supplementary file 5 [file EC-26-0247_supplementary_figure_5.pdf]

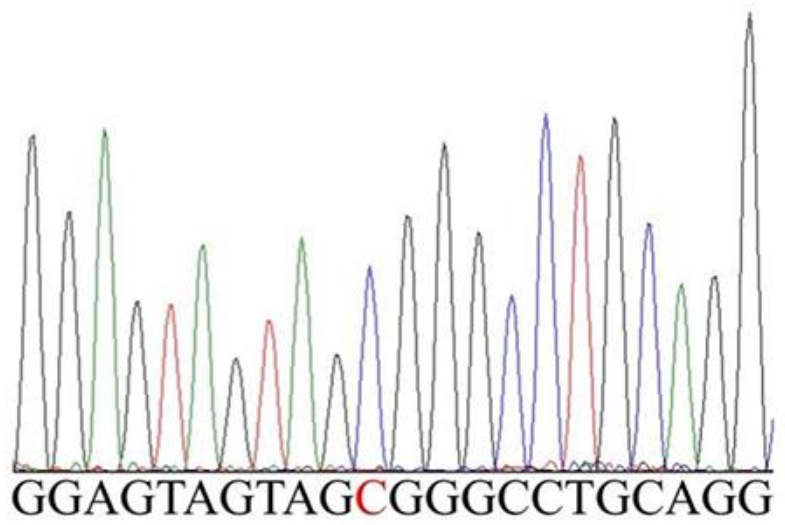

POR c.1370G>A (p.R457H) wildtype GGAGTAGTAGCGGGCCTGCAGG
