# Supplementary material for: Clinical and genetic spectrum of cytochrome P450 oxidoreductase deficiency in Chinese patients
Source: Endocr Connect. 2026 Sep 8;15(9):e260247. doi: 10.1530/EC-26-0247 (PMC13588290; doi:10.1530/EC-26-0247)
Supplement: Supplementary file 6 [file EC-26-0247_supplementary_figure_6.pdf]

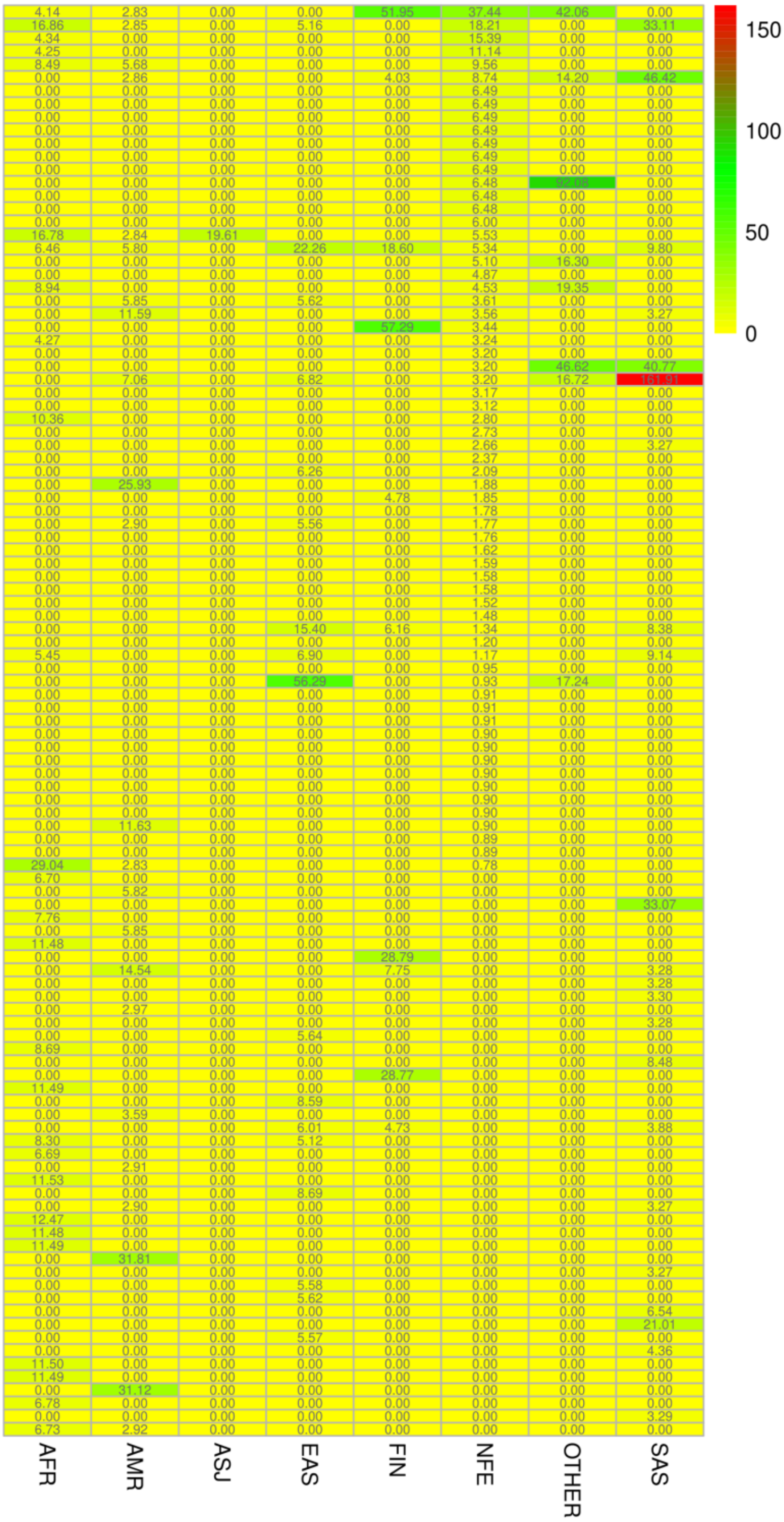
AFR
AMR
ASJ
EAS
FIN
NFE
OTHER
SAS
150
100
50
0
